# Automated Detection and Segmentation of Ascending Aorta Dilation on a Non-ECG-Gated Chest CT Using Deep Learning

**DOI:** 10.3390/diagnostics15182336

**Published:** 2025-09-15

**Authors:** Fargana Aghayeva, Yusuf Abdi, Ahmad Uzair, Ayaz Aghayev

**Affiliations:** 1 Department of Radiology, Brigham and Women’s Hospital, Mass General Brigham, Boston, MA 02115, USA; faghayeva@bwh.harvard.edu; 2 Khoury College of Computer Sciences, Northeastern University, Boston, MA 02115, USA; yuuabdi@gmail.com; 3 College of Computing and Information Technology, University of Doha for Science and Technology, Doha P.O. Box 24449, Qatar; uzair.ahmad@udst.edu.qa; 4 Harvard Medical School, Boston, MA 02115, USA

**Keywords:** deep learning, CNN, U-Net, aortic dilation, non-ECG-gated CT

## Abstract

**Background/Objectives:** Ascending aortic (AA) dilation (diameter ≥ 4.0 cm) is a significant risk factor for aortic dissection, yet it often goes unnoticed in routine chest CT scans performed for other indications. This study aimed to develop and evaluate a deep learning pipeline for automated AA segmentation using non-ECG-gated chest CT scans. **Methods:** We designed a two-stage pipeline integrating a convolutional neural network (CNN) for focus-slice classification and a U-Net-based segmentation model to extract the aortic region. The model was trained and validated on a dataset of 500 non-ECG-gated chest CT scans, encompassing over 50,000 individual slices. **Results:** On the held-out test set (10%), the model achieved a Dice similarity coefficient (DSC) score of 99.21%, an Intersection over Union (IoU) of 98.45%, and a focus-slice classification accuracy of 98.18%. Compared with traditional rule-based and prior CNN-based methods, the proposed approach achieved markedly higher overlap metrics while maintaining low computational overhead. **Conclusions:** A lightweight CNN+U-Net deep learning model can enhance diagnostic accuracy, reduce radiologist workload, and enable opportunistic detection of AA dilation in routine chest CT imaging.

## 1. Introduction

Ascending aortic (AA) dilation is a major risk factor for aortic dissection, a life-threatening condition. However, ascending aortic dilation is frequently under-recognized in routine chest CT scans, as the primary clinical focus is often directed toward alternative indications, such as pneumonia. Deep learning, particularly convolutional neural networks (CNNs), has revolutionized medical imaging by enabling automated segmentation and measurement of anatomical structures with high accuracy [1]. These models excel at extracting complex hierarchical and spatial features directly from raw imaging data, making them particularly effective for segmentation and measurement tasks. Their ability to analyze vast datasets with precision has transformed how clinicians approach diagnostic imaging, reducing human effort and variability.

CNNs have been successfully applied in organ segmentation tasks such as liver segmentation [2], brain tumor detection [3], and cardiac imaging [4]. One notable success is the use of U-Net in retinal vessel segmentation, which significantly improved segmentation accuracy [5]. The U-Net architecture, originally designed for biomedical imaging, has become the gold standard due to its encoder–decoder structure with skip connections, which preserve spatial details and enhance segmentation accuracy.

Deep learning has been applied to opportunistic disease detection in chest CT scans, particularly for lung nodules, pulmonary embolism, and coronary artery calcifications. However, its application to aortic dilation detection in non-ECG-gated CT scans remains largely unexplored.

Definition. In this study, segmentation refers to the automated delineation of the ascending aorta on each CT slice, producing a binary mask that distinguishes aortic pixels from surrounding tissues. This mask enables objective measurement of aortic size.

The ascending aorta, originating from the heart’s left ventricle, is the largest artery in the human body and plays a pivotal role in distributing oxygen-rich blood to the systemic circulation. Its dilation, defined as a diameter of ≥ 4.0 cm, is associated with severe complications, including aortic dissection and rupture [6]. These conditions, if undiagnosed or left untreated, can lead to life-threatening events, with mortality rates exceeding 31% in cases of acute aortic dissection [6]. Recent studies estimate that incidental aortic dilations are present in 2.7–23% of routine chest CT scans, yet many of these cases may go unrecognized or undocumented, potentially due to the absence of standardized measurement protocols or automated detection tools [7]. This wide range reflects heterogeneity across studies in population (screening vs. symptomatic cohorts), imaging protocols (contrast use), and threshold definitions (fixed ≥ 4.0 cm vs. body-size-indexed cutoffs), as well as reporting practices.

Clinical Gap: The ideal method for evaluating the ascending aorta is ECG-gated Computed Tomography Angiography (CTA), which requires specialized imaging technology and trained personnel. However, the ascending aorta is visible on non-gated chest CT scans, which are primarily performed for other indications, such as lung cancer screening or the evaluation of pulmonary conditions. With approximately 13 million non-gated chest CT scans conducted annually in the U.S. [7], these images present an underutilized opportunity for assessing the ascending aorta. Given that aortic abnormalities may be overlooked due to the primary focus on other pathologies, deep learning-driven analysis could serve as a valuable tool to opportunistically detect aortic abnormalities, enhancing early diagnosis and patient outcomes.

What Others Have Been Doing: Prior research has focused on aortic segmentation using rule-based or machine learning models designed for ECG-gated CT angiography [8]. While 3D U-Net models have demonstrated success in gated imaging datasets, they often suffer from poor generalizability when applied to non-gated scans, which contain greater motion artifacts and variable contrast conditions [9]. Some approaches use motion-compensation techniques or hybrid CNN–transformer models [10], but their computational complexity limits their clinical usability.

Is This Unique to Our Solution? Unlike previous approaches that rely on ECG-gated imaging or full 3D U-Net segmentation, our method integrates a CNN-based focus-slice classification module to efficiently select the most relevant CT slice and a U-Net-based segmentation model to extract the ascending aorta region. This hybrid approach minimizes computational overhead while improving segmentation accuracy.

**Figure 1:**
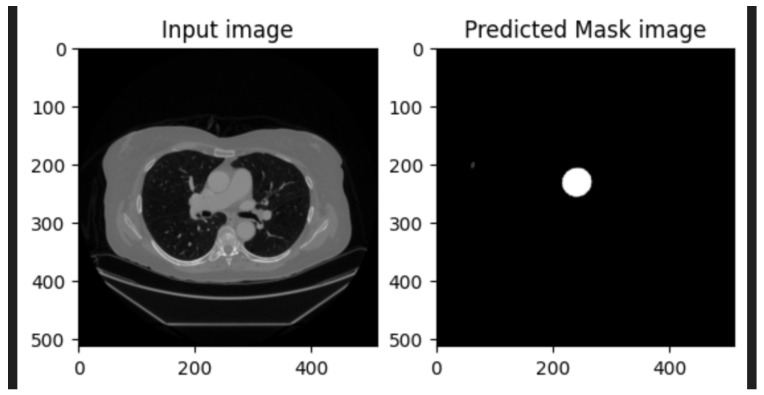
Example of CNN-based focus-slice classification and U-Net segmentation results. The CNN model identifies the most relevant focus slice containing the largest ascending aorta, while the U-Net model accurately segments the aortic region.

Comparison with Existing Methods: Traditional rule-based segmentation methods report a Dice score of 78.3%, while prior CNN-based methods report Dice scores of 85.6% (basic CNN segmentation) and 90.5% (3D U-Net) [11]. On our held-out test set, the proposed method attains a Dice score of 99.21% and an IoU of 98.45%, representing an 8.7–20.9 percentage-point improvement in the Dice score and a 16.4–33.1 percentage-point improvement in the IoU over reported baselines. An illustrative example of CNN-based focus-slice classification and subsequent U-Net segmentation of the ascending aorta is shown in Figure 1.

Key Takeaways: This study presents a lightweight CNN+U-Net pipeline for automated detection and segmentation of ascending aortic dilation on non-ECG-gated chest CT scans. The method achieves excellent performance, with a Dice score of 99.21%, an IoU of 98.45%, and a slice-classification accuracy of 98.18% on the test set. By integrating focus-slice selection with segmentation, the model reduces computational overhead while maintaining superior accuracy compared to rule-based and prior CNN-based methods. This approach enhances diagnostic efficiency, supports radiologists in clinical decision-making, and enables opportunistic detection of aortic dilation in routine imaging.

## 2. Related Works

Segmentation of medical images, particularly for the ascending aorta, has been a focus of extensive research due to its critical role in diagnosing cardiovascular diseases. Traditional approaches for aortic segmentation rely on manual or semi-automated methods, which involve manual contouring by radiologists or rule-based techniques. While these approaches can provide reasonable accuracy, they are time-intensive, subject to inter-observer variability, and prone to inconsistencies, especially in datasets with motion artifacts [12].

**Figure 2:**
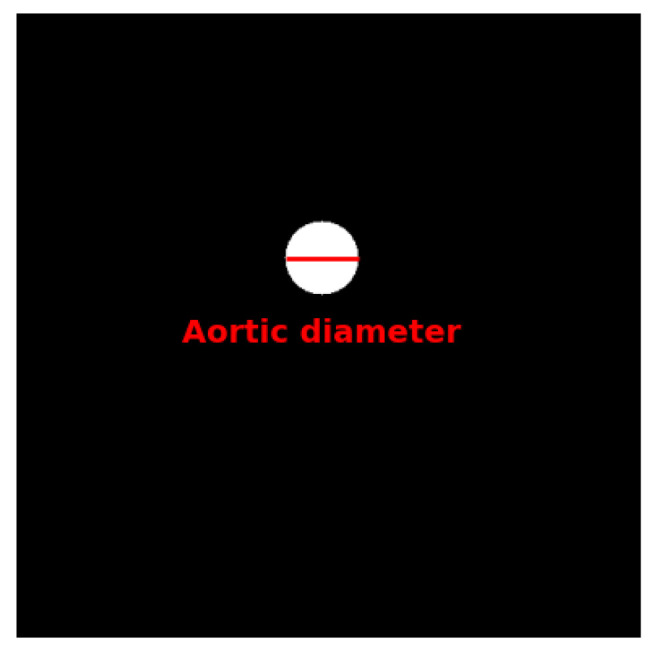
Example of U-Net segmentation applied to medical imaging, illustrating aortic diameter measurement.

Recent advancements in deep learning have significantly transformed medical-image analysis. Convolutional neural networks (CNNs) have been widely adopted for organ segmentation, lesion detection, and feature extraction [1,13]. For instance, methods like VGGNet and ResNet have demonstrated their efficacy in capturing hierarchical image features, which are crucial for complex medical imaging tasks [14]. Additionally, fully convolutional networks (FCNs) have been leveraged to adapt CNNs for pixel-wise segmentation tasks, laying the groundwork for more advanced architectures like U-Net [5]. An example of U-Net segmentation applied to medical imaging, illustrating aortic diameter measurement, is shown in Figure 2.

U-Net, originally proposed for biomedical-image segmentation, has become the gold standard for many medical imaging applications. Its encoder–decoder structure, combined with skip connections, allows it to effectively capture both low-level and high-level features, enabling precise segmentation even with limited training data. U-Net and its variants have been successfully applied to tasks such as brain tumor segmentation, liver segmentation, and cardiac MRI analysis [3,4]. However, existing U-Net-based methods are typically designed for ECG-gated CT scans, which inherently have fewer motion artifacts, limiting their direct applicability to non-ECG-gated scans [9].

### 2.1. Limitations of Existing Aortic Segmentation Methods

Techniques addressing segmentation in non-ECG-gated chest CT scans remain sparse. Some studies have attempted to use motion-correction algorithms or atlas-based registration methods to reduce artifacts in non-gated scans [15]. While these methods offer improvements, they are computationally intensive and often fail in cases with severe motion artifacts or atypical anatomical variations.

### 2.2. Deep Learning Approaches for Non-Gated Chest CT

Deep learning-based pipelines tailored for non-ECG-gated scans have recently emerged, aiming to bridge this gap. Examples include the following:

- Hybrid CNN approaches: These integrate motion-artifact detection with segmentation tasks, but they often require pre-labeled motion-artifact datasets, making them less scalable [6].
- Attention-based U-Nets: By incorporating attention mechanisms, these models improve focus on relevant regions. However, their performance can be heavily reliant on the quality of the training data and annotations [16].

**Figure 3:**
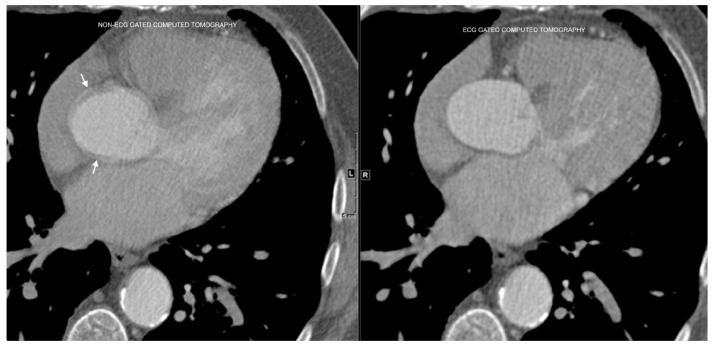
Comparison of ECG-gated and non-ECG-gated chest CT scans of the aorta. Arrows denote the blurry borders of the ascending aorta in non-ECG-gated images, which appear sharper in ECG-gated images. Motion artifacts in non-ECG-gated scans pose significant challenges in segmentation tasks.

The differences between ECG-gated and non-ECG-gated chest CT scans, and the associated motion artifacts, are illustrated in Figure 3.

### 2.3. Proposed Framework for Non-Gated Aortic Segmentation

The proposed method differentiates itself by combining CNN-based focus-slice classification with a U-Net segmentation framework. The focus-slice classification step identifies the most relevant slices, significantly reducing irrelevant slices passed to the segmentation stage. This approach not only addresses the motion-artifact issue but also optimizes computational efficiency by narrowing the analysis to the most informative slices. Furthermore, the U-Net model is trained using a combination of Dice loss and binary cross-entropy loss, ensuring both pixel-wise accuracy and region overlap.

Unlike existing methods, this pipeline was specifically designed for non-ECG-gated chest CT scans, leveraging advanced data augmentation techniques to enhance robustness across diverse datasets. The combination of these innovations establishes a reliable and efficient framework for accurate segmentation of the ascending aorta, overcoming many limitations of previous methods.

## 3. Methodology

The proposed pipeline for ascending aorta segmentation combines preprocessing, focus-slice classification using CNNs, and segmentation using a U-Net architecture. Each component is designed to address the unique challenges of non-ECG-gated chest CT scans, including motion artifacts and irrelevant slices.

### 3.1. Pipeline Overview

The pipeline consists of three main stages:

1. Preprocessing: Image normalization, resizing, and artifact reduction.
2. Focus-slice classification: A CNN model detects slices containing the ascending aorta and filters out irrelevant slices.
3. Segmentation: A U-Net model performs precise segmentation on the selected slices.

**Figure 5:**
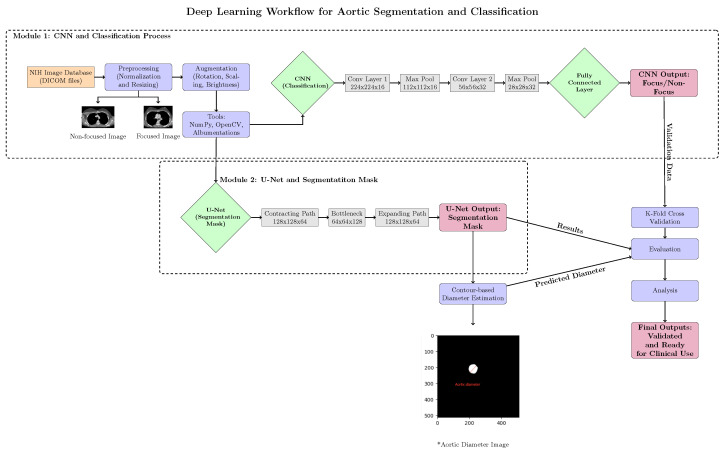
Overview of the proposed pipeline for ascending aorta segmentation. The three main stages are preprocessing, focus-slice classification, and segmentation. The asterisk (*) denotes the aortic diameter image used for contour-based diameter estimation.

Figure 5 provides a detailed overview of the pipeline.

### 3.2. Preprocessing

Preprocessing ensures data consistency and reduces artifacts. First, pixel intensities were normalized to [0,1] using $I_{\mathrm{norm}}=\frac{I-I_{\min}}{I_{\max}-I_{\min}}$ , where *I* denotes the pixel value and $I_{\min},I_{\max}$ are the minimum and maximum intensities in the image. All slices were then resized to a uniform size of 256×256 . Finally, Gaussian smoothing and non-local means filtering were applied to suppress noise while preserving edges.

### 3.3. Focus-Slice Classification

The CNN model detects slices containing the ascending aorta while filtering out irrelevant slices that do not contribute to segmentation. This classification reduces computational overhead and enhances segmentation accuracy by ensuring only the most relevant images are processed. The architecture includes the following:

- Convolutional layers: Extract hierarchical spatial features using 3×3 filters with ReLU activation.
- Pooling layers: Reduce spatial dimensions using 2×2 max-pooling.
- Fully connected layers: Aggregate features for binary classification with softmax activation.

The binary cross-entropy loss is used for optimization:

$$L_{\mathrm{BCE}}=-\frac{1}{N}\sum_{i=1}^{N}\left[y_{i}\log\left({\hat{y}}_{i}\right)+\left(1-y_{i}\right)\log\left(1-{\hat{y}}_{i}\right)\right]$$

where $y_{i}$ is the ground-truth label and ${\hat{y}}_{i}$ is the predicted probability.

### 3.4. Segmentation

The U-Net architecture performs segmentation on selected slices:

- Contracting path: Sequential convolutional and max-pooling layers extract features at multiple scales.
- Bottleneck: Captures the most abstract features in the data.
- Expanding path: Transposed convolutions and skip connections restore resolution and merge low-level and high-level features.

The Dice loss optimizes segmentation performance:

$$L_{\mathrm{Dice}}=1-\frac{2\sum_{i=1}^{N}p_{i}g_{i}}{\sum_{i=1}^{N}p_{i}^{2}+\sum_{i=1}^{N}g_{i}^{2}}$$

where $p_{i}$ and $g_{i}$ are the predicted and ground-truth masks. A combined loss function is used:

$$L=L_{\mathrm{BCE}}+\lambda L_{\mathrm{Dice}}$$

where λ balances pixel-wise accuracy and overlap.

### 3.5. Hybrid CNN Variants (Classification–Segmentation and Attention)

Beyond the baseline U-Net segmenter, we considered hybrid designs that couple a global classification signal with the segmentation backbone and/or augment the encoder with attention modules. First, we attach a binary classification head at the encoder bottleneck to predict dilation status (diameter ≥ 4.0 cm) using global average pooling followed by a linear layer and sigmoid. The network is optimized with a joint objective:

$$L_{\mathrm{total}}=L_{\mathrm{seg}}+\alpha \;L_{\mathrm{cls}},$$

where $L_{\mathrm{seg}}=L_{\mathrm{BCE}}+\lambda \;L_{\mathrm{Dice}}$ (as defined above); $L_{\mathrm{cls}}$ is binary cross-entropy for the dilation label; and α balances the tasks. The classification signal can regularize features and encourage shape-consistent embeddings that benefit boundary delineation.

Second, we considered encoder enhancements with lightweight attention: squeeze-and-excitation (SE) channel reweighting and a convolutional block attention module (CBAM) to modulate salient channels and spatial regions [17,18]. It should also be noted that attention-gated U-Net and UNet++ skip re-designs can improve multi-scale aggregation [19,20]. Additionally, a 2.5D input mode (stacking adjacent slices as channels) can stabilize boundaries under motion without the full computational cost of 3D. These variants are architecturally compatible with our pipeline and will be evaluated in follow-up experiments.

### 3.6. Data Augmentation

Data augmentation enhances robustness and generalizability by creating synthetic variations of the training data, preventing overfitting, and improving model generalization to unseen cases. The applied augmentations include the following:

- Geometric transformations: Random rotations ( $\pm 20^{\circ }$ ), flips, and scaling to simulate variations in patient positioning.
- Intensity adjustments: Random brightness and contrast variations to account for different CT scanner settings.
- Elastic deformations: Mimic anatomical variability by applying smooth distortions, improving model robustness to structural differences.
- Gaussian noise: Adds random pixel intensity variations to simulate acquisition noise and improve model tolerance to lower-quality scans.

All transformations were applied to images and masks jointly and kept within conservative, label-preserving ranges consistent with variability seen on routine non-ECG-gated chest CT. Augmentations were applied only to the training set; the validation and test subsets were not augmented.

**Figure 4:**
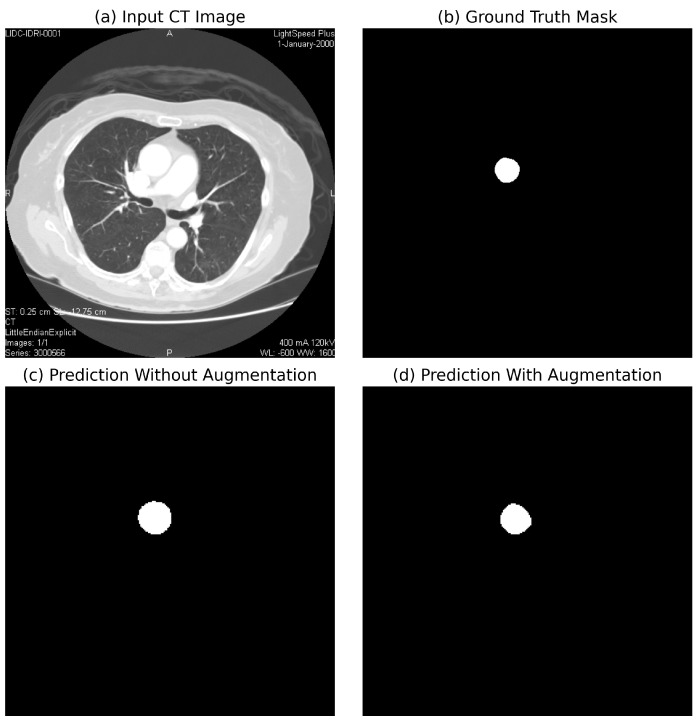
Comparison of segmentation performance with and without data augmentation. Augmentation significantly improves model generalization and accuracy.

Figure 4 compares segmentation performance with and without data augmentation.

Impact of Augmentation: To quantify the effect of augmentation, we compared segmentation performance with and without augmentation. The results indicated a 6.8 percentage-point improvement in the Dice score (86.4% vs. 93.2%) and a 4.3 percentage-point improvement in the IoU (84.2% vs. 88.5%) when augmentation was applied, highlighting its critical role in training deep learning models.

### 3.7. Optimization

The Adam optimizer adjusts learning rates adaptively:

$$m_{t}=\beta_{1}m_{t-1}+\left(1-\beta_{1}\right)g_{t},\;v_{t}=\beta_{2}v_{t-1}+\left(1-\beta_{2}\right)g_{t}^{2}$$

$$\theta_{t}=\theta_{t-1}-\frac{\alpha \cdot m_{t}}{\sqrt{v_{t}}+\epsilon }$$

where $m_{t}$ and $v_{t}$ are gradient estimates, $\theta_{t}$ are model parameters, α is the learning rate, and ϵ is a small constant. Training was performed for 100 epochs with early stopping.

### 3.8. Pipeline Flowchart

The overall workflow of our proposed method is illustrated in Figure 5. It consists of three main steps: preprocessing, focus-slice classification, and segmentation.

## 4. Experimental Setup

This section describes the dataset, annotation processes, inclusion and exclusion criteria, and the hardware and software environment used for training and evaluating the models.

### 4.1. Dataset Characteristics

Data were obtained from the National Lung Screening Trial (NLST) via the NCI Cancer Data Access System (CDAS) [21]. All images were de-identified, and details regarding anonymization and data use are provided in the ethics statement. The dataset comprised 500 non-ECG-gated chest CT scans collected across multiple institutions to ensure diversity and robustness. Each scan was annotated by expert radiologists with more than ten years of experience. While the original scans varied in resolution, all images were resampled to a uniform size of 256×256 pixels during preprocessing. Depending on the scanning protocol, the number of slices per scan ranged from approximately 100 to 300.

### 4.2. Annotation Process

Manual annotation was carried out on all slices containing the ascending aorta using specialized software. Radiologists first identified the region of interest by manually segmenting the ascending aorta with a polygonal tool. The radiologists then reviewed the segmentations to ensure accuracy and consistency. The final segmentations were converted into binary masks, in which voxels within the aortic region were labeled as one and all other voxels as zero.

### 4.3. Inclusion and Exclusion Criteria

Scans were included if they were non-ECG-gated chest CT studies of sufficient image quality and provided complete coverage of the thoracic region, including the ascending aorta. Scans were excluded if they contained severe motion artifacts, lacked full thoracic coverage, or followed imaging protocols that deviated significantly from standard practices.

### 4.4. Training and Validation Setup

The dataset was divided into training, validation, and testing subsets, with 400 scans (80%) used for training, 50 scans (10%) for validation, and 50 scans (10%) for final evaluation. To enhance model robustness, extensive data augmentation techniques were applied, including geometric transformations (rotations and flips), intensity modifications, and elastic deformations. Model optimization employed a combined loss function consisting of binary cross-entropy loss and Dice loss, as described in Section 2.

### 4.5. Hardware and Software Environment

All experiments were conducted in a high-performance computing environment equipped with an NVIDIA Tesla V100 GPU (32 GB VRAM), an Intel Xeon Gold 6230 CPU (2.1 GHz, 20 cores), and 256 GB of system memory. The models were implemented in PyTorch (version 1.12.1) and executed on Ubuntu 20.04 LTS. ITK-SNAP (version 3.8.0) and MATLAB (R2022a) were used for generating binary masks, while data preprocessing and augmentation were carried out using NumPy (version 1.23), OpenCV (version 4.6), and Albumentations (version 1.3.0).

## 5. Results

### 5.1. Quantitative Metrics

Unless otherwise noted, all metrics reported below are from the held-out test subset (10%). Training/validation curves are shown elsewhere only to illustrate optimization dynamics and potential overfitting. Table 1 summarizes the performance of the focus-slice classifier on the held-out test set.

Table 2 reports the segmentation performance on the test set, presented on a slice-wise basis.

### 5.2. Precision–Recall and ROC Curves

**Figure 6:**
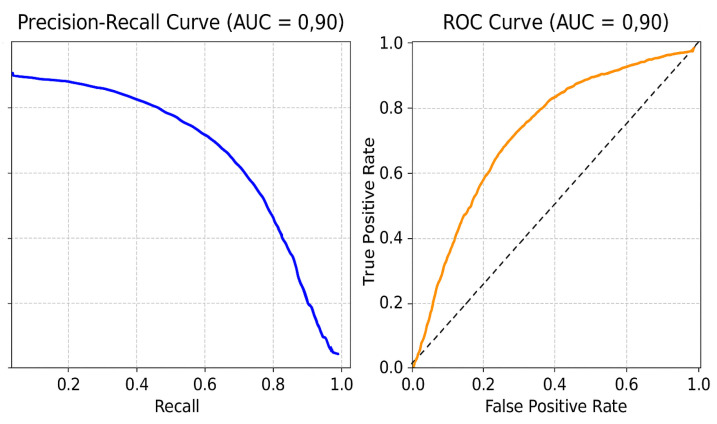
Precision–recall and ROC curves of the CNN-based focus-slice classification model.

Figure 6 presents the precision–recall and ROC curves for the CNN-based focus-slice classification model. These plots emphasize the high precision and recall achieved, underscoring the robustness of the model in identifying relevant slices.

### 5.3. Parameter Optimization Analysis

**Figure 7:**
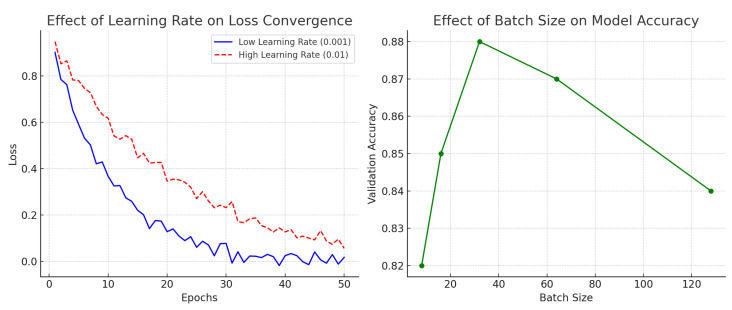
Parameter optimization analysis: impact of the learning rate and batch size on model accuracy and loss convergence.

Figure 7 illustrates the effect of the learning rate and batch size on model performance. The left panel demonstrates that a lower learning rate ( 0.001 ) resulted in smoother and more stable convergence of the loss function, ultimately reaching near-zero values after 50 epochs. In contrast, a higher learning rate ( 0.01 ) resulted in unstable convergence, with larger fluctuations and a slower reduction in loss.

The right panel shows the impact of batch size on validation accuracy. Accuracy increased substantially when moving from a batch size of 8 to 32, achieving a peak at a batch size of 32 (approximately 0.88). Beyond this point, performance gradually declined, with batch sizes of 64 and 128 yielding reduced accuracy. These results suggest that moderately sized batches provide an optimal balance between stability and generalization, while excessively large batches degrade performance due to limited stochasticity in gradient updates.

### 5.4. Qualitative Analysis

**Figure 8:**
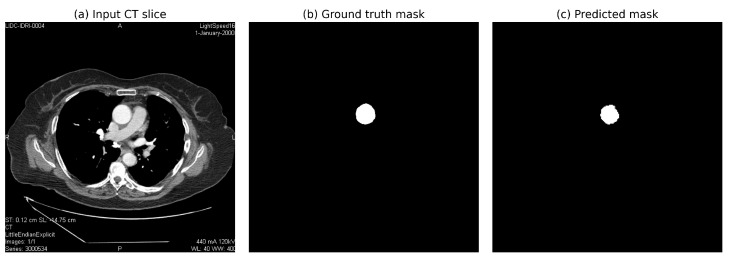
Sample segmented images of the ascending aorta: (**a**) input CT slice; (**b**) true mask, (**c**) predicted mask generated by the U-Net model.

Figure 8 illustrates the segmentation output of the proposed pipeline, demonstrating the U-Net model’s ability to accurately segment the ascending aorta from non-ECG-gated CT slices. The predicted mask highlights precise segmentation despite motion artifacts and anatomical variations.

### 5.5. Comparative Analysis

The proposed framework was compared with existing methods, as shown in Table 3. The results indicate that our approach achieved strong overlap with ground-truth annotations, reflected in the higher Dice and IoU values. (The values listed in the table for the “proposed framework” are from our test set.)

The improvement in segmentation accuracy can be attributed to the integration of CNN-based focus-slice selection with a U-Net-based segmentation model. Compared to the 3D U-Net approach [11], our framework achieved higher Dice and IoU values, indicating better spatial alignment with the ground truth. Traditional rule-based approaches [22] may suffer from inter-observer variability and limited adaptability to complex imaging variations, while basic CNN-based segmentation [23] can struggle with generalization. Our framework addresses these limitations by combining classification for optimal slice selection with U-Net segmentation. To the best of our knowledge, there are no prior studies that directly report ascending aorta segmentation on the NLST dataset. However, related thoracic CT benchmarks using nnU-Net and TotalSegmentator have demonstrated strong performance, with Dice scores ranging from approximately 0.78 for lung-lesion segmentation to 0.94 for multi-organ CT segmentation [24,25]. While not directly comparable, these works highlight the robustness of nnU-Net in thoracic imaging and provide a useful context for our results, which achieve higher Dice and IoU values on the targeted ascending aorta segmentation task.

## 6. Discussion

### 6.1. Key Findings

**Figure 9:**
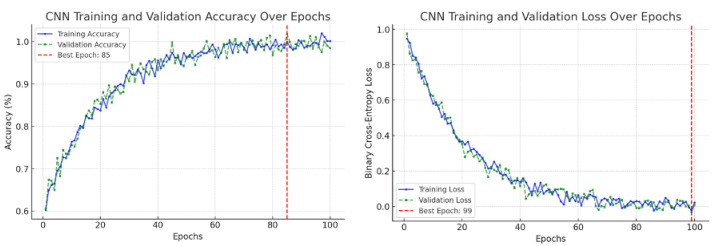
CNN training over 100 epochs. Accuracy approaches a plateau, while binary cross-entropy loss decreases, consistent with convergence.

**Figure 10:**
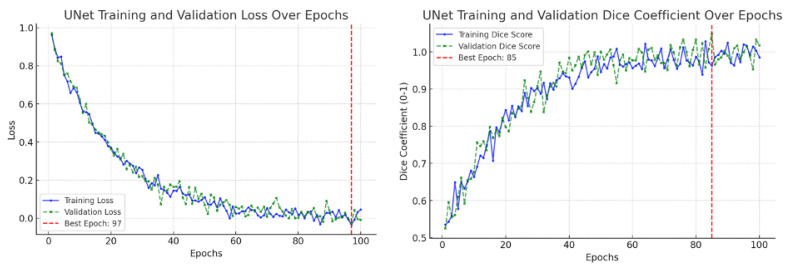
U-Net training over 100 epochs. Training loss and validation loss decline as the Dice coefficient rises and stabilizes.

This study confirmed the feasibility of using deep learning for automated segmentation of the ascending aorta in non-ECG-gated CT. Training curves are a standard diagnostic tool in deep learning, showing the evolution of model performance during optimization [26]. Typically, the training loss indicates how well the model fits the training data, while the validation loss measures generalization to unseen data. Similarly, the training accuracy measures correct predictions on the training set, and the validation accuracy reflects performance on held-out data. These graphs are used to detect problems such as underfitting (both training and validation performance remain poor), overfitting (training improves but validation stagnates or worsens), or unstable optimization (losses oscillate without convergence) [27,28]. In our results, the CNN focus-slice classifier showed decreasing training and validation losses with validation accuracy plateauing, indicating stable generalization without overfitting (Figure 9). The U-Net segmenter demonstrated steadily declining losses and a rising Dice coefficient, which stabilized near convergence (Figure 10). Using a combined binary cross-entropy loss and Dice loss further stabilized optimization and improved overlap quality. Moreover, data augmentation improved segmentation performance, yielding closer alignment with the ground truth compared to the non-augmented model (Figure 4), thereby supporting the clinical validity of our augmentation pipeline.

Based on the graph outcomes, the model demonstrated high segmentation accuracy, even in the presence of varying anatomical structures. The combined loss function (binary cross-entropy + Dice loss) significantly improved training stability and performance.

### 6.2. Impact on Radiologist Expertise

A common concern is whether automated segmentation and related deep learning tools may cause the traditional interpretive skills of radiologists to “atrophy.” We emphasize that our framework is designed as a decision-support system rather than a replacement for radiologists. Automated segmentation reduces the time and variability associated with manual contouring, allowing radiologists to focus on higher-level interpretive tasks such as correlating imaging findings with clinical presentation, integrating multi-modality data, and making patient-centered management decisions. In this way, deep learning serves to augment radiologist performance and efficiency, not diminish core diagnostic expertise.

### 6.3. Clinical Validity of Data Augmentation

Our augmentation choices were selected to mirror routine variability in non-ECG-gated chest CT while preserving aortic anatomy. In-plane rotations (within $\pm 20^{\circ }$ ) approximate patient positioning differences and modest gantry tilt; when applied jointly to the image and its mask, this range preserves the circular/elliptical cross-section of the ascending aorta and does not distort diameters. In-plane flips are label-preserving for a segmentation task focused on the aortic contour (not laterality), and small isotropic scalings model differences in field-of-view and pixel spacing encountered across scanners and breath-holds; final diameter measurements are computed on non-augmented images at native spacing.

Low-magnitude brightness/contrast jitter reflects scanner, kernel, and dose variations commonly observed in practice. Mild, smooth elastic deformations emulate patient-specific anatomical variability and non-ECG-gated motion while avoiding topology-breaking warps. Additive Gaussian noise models acquisition noise, particularly in lower-dose protocols. These operations are standard, label-preserving augmentations for medical-image segmentation and are reported to improve robustness to clinical variability [1,9]. Consistent with this, our ablation (Figure 4) showed higher Dice and IoU values with augmentation on this dataset.

### 6.4. Error Analysis

Despite achieving high accuracy, certain error cases were observed. These errors were primarily caused by imaging artifacts, anatomical variations, and limitations in scan resolution. Motion artifacts, for example, can result in blurred segmentation boundaries, making precise localization of the aortic structure challenging. Additionally, variations in contrast levels across different CT scans may affect the model’s ability to consistently differentiate the aortic wall from surrounding tissues. Table 4 summarizes common segmentation errors and their potential causes.

A frequent anatomical variant is the so-called “bovine aortic arch,” where the brachiocephalic and left common carotid arteries share a common origin. This variation does not typically confuse the proposed system because our pipeline segments the ascending aorta based on cross-sectional shape and continuity rather than branch vessel anatomy. However, the presence of closely adjacent branch vessels may introduce minor boundary ambiguity in some slices. These cases were correctly segmented in our dataset, suggesting that the model generalizes well to such anatomical variants. Future extensions could explicitly incorporate branch-level annotations to further validate robustness across anatomical subtypes.

Hybrid classification–segmentation models and attention-augmented encoders (SE/CBAM, attention-gated U-Net, UNet++) are natural extensions for non-ECG-gated scans, where motion and contrast variability challenge boundary fidelity. A multi-task classification head supplies a global dilation signal that can regularize the encoder features used by the segmenter, while 2.5D inputs may improve through-plane consistency at a modest cost. We plan a systematic ablation of these variants in a multi-center setting [17,18,19,20].

### 6.5. Limitations and Future Directions

The current pipeline segments the ascending aorta beginning above the sinotubular junction and therefore does not explicitly measure the aortic root. In clinical practice, root diameters on gated chest CTA are measured using multiplanar reformations with several conventions (e.g., sinus-to-sinus or commissure-to-sinus). Multiplanar reformatting on routine, non-gated chest CT is not feasible; accordingly, root-specific measurements are outside the scope of this study.

While the proposed pipeline demonstrated strong performance, several limitations remain. Motion artifacts are a recurring failure mode; incorporating spatiotemporal attention or sequence-consistency constraints may improve robustness. Generalizability would benefit from training and external validation on multi-center, multi-vendor cohorts. Moving from a 2D U-Net toward 2.5D/3D segmentation could enhance volumetric consistency and boundary fidelity.

## 7. Conclusions and Future Work

The proposed method achieves strong test-set performance (classifier accuracy: 98.18%; Dice score: 99.21%; IoU: 98.45%) and, in practice, improves segmentation in the presence of motion, reduces manual workload by automating slice selection and contouring, and yields reproducible contours. All metrics are reported on a held-out test set. Looking ahead, we will pursue multi-center external validation, explore 2.5D/3D segmentation to enhance volumetric consistency, and evaluate an end-to-end, clinician-in-the-loop workflow that integrates focus-slice classification, segmentation, anomaly detection, and quality assurance/uncertainty checks. We will also evaluate hybrid classification–segmentation variants and attention-augmented encoders (SE/CBAM, UNet++) to further improve boundary fidelity in non-ECG-gated scans. We also plan to incorporate explainable AI (e.g., Grad-CAM) to increase radiologists’ confidence and refine clinical usability.

## Figures and Tables

**Table 1:** Focus-slice classifier performance on the test set (N = 769 slices; 70 positive).

Metric	Value
Accuracy (%)	98.18
Precision (focus)	0.9103
Recall/sensitivity (focus)	1.0000
False positive rate (FPR)	2.23%
ROC AUC (focus)	0.9992

**Table 2:** Segmentation performance on the test set (slice-wise).

Metric	Mean	Median
Dice	0.9921	0.9925
IoU	0.9845	0.9852

**Table 3:** Comparative analysis with existing methods (higher is better).

Method	Dice Score (%)	IoU (%)
Traditional rule-based [22]	78.3	65.4
Basic CNN segmentation [23]	85.6	72.8
3D U-Net [11]	90.5	82.1
Proposed framework (test)	99.21	98.45

**Table 4:** Error analysis: potential causes and observed effects.

Error Type	Potential Cause	Observed Effect
Motion artifacts	Patient movement during scans	Blurry segmentation boundaries
Small aortic diameters	Limited resolution	Reduced Dice scores for small regions
High-contrast variations	Non-uniform imaging conditions	Over-segmentation in certain areas

## Data Availability

Data were obtained from the National Lung Screening Trial (NLST) via the NCI Cancer Data Access System (CDAS) [21]. All images are de-identified; details of data use and anonymization are provided in the ethics statement. The dataset is publicly available at https://cdas.cancer.gov/learn/nlst/images/ (accessed on 22 November 2023) Some preliminary results of this work were presented at the Radiological Society of North America (RSNA) Annual Meeting 2024 [29].
